# Is Intravenous Heparin a Contraindication for TPA in Ischemic Stroke?

**DOI:** 10.1155/2017/9280961

**Published:** 2017-02-05

**Authors:** Zain Kulairi, Nisha Deol, Renee Tolly, Rohan Manocha, Maliha Naseer

**Affiliations:** Department of Internal Medicine, Crittenton Hospital Medical Center, Wayne State University, Rochester, MI 48307, USA

## Abstract

There are approximately 2 million cardiac catheterizations that occur every year in the United States and with an aging population this number continues to rise. Adverse events due to this procedure occur at low rates and include stroke, arrhythmia, and myocardial infarctions. Due to the high volume of procedures there are a growing number of adverse events. Stroke after cardiac catheterization (SCC) has an incidence between 0.27 and 0.5% and is one of the most debilitating complications leading to high rates of mortality and morbidity. Given the relatively uncommon clinical setting of stroke after cardiac catheterization, treatment protocols regarding the use of IV or IA thrombolysis have not been adequately developed. Herein, we describe a case of a 39-year-old male who developed a stroke following a cardiac catheterization where IV thrombolysis was utilized although the patient was on heparin prior to cardiac catheterization.

## 1. Background

The number of cardiac catheterizations performed every year is increasing, and due to this it is important to know the adverse events associated with the procedure and more importantly how to deal with these events. Stroke is a potential complication and management can be complicated due to recent catheterization. In a study of 20,679 patients where SCC occurred in 0.30% of participants, the most common neurologic manifestations were motor or speech deficits with the combination of unresponsiveness or altered mental status occurring in 45% of all patients who experienced a stroke [1]. According to Hamon et al. [2], patients who developed a stroke after PCI were more likely to also have underlying diabetes mellitus, hypertension, history of prior stroke, and/or renal failure.

Management of stroke plays a crucial role as there is increased mortality seen in patients with CVA. The study done by Dukkipati et al. in 2004 was significant for death in 25% of those with CVA, compared with 1.5% of those without CVA (*P* < 0.0001). The same study showed 69% of patients with hemorrhagic stroke and 21% of patients with ischemic stroke died in the hospital. Early recognition of neurological deficits following cardiac catheterization allows for an opportunity for intervention with thrombolytic therapy. However, the use of heparin and a prolonged aPTT are contraindications to tPA [3] and therefore may require frequent monitoring within the window for tPA.

## 2. Case Report

This is a 39-year-old male with a past medical history of coronary artery disease requiring coronary artery bypass graft in 2009 and percutaneous coronary intervention and stenting in 2008 to the left anterior descending artery and right coronary artery. Patient also has hypercholesterolemia and hypothyroidism. He presented to the emergency department complaining of substernal chest pain associated with dizziness and shortness of breath. This occurred once to two times a week. His last stress test was in 2013 which was insignificant. One month ago he saw his cardiologist who recommended using nitroglycerin more frequently. His chest pain initially improved but for the past week his pain was increasing in severity and radiating to the left shoulder. Patient denied any nausea, vomiting, sweating, or loss of consciousness. The chest pain was not aggravated or alleviated by position or medication. Vital signs were within normal limits. Physical examination was normal including cardiac and neurological exam. The patient was seen by the cardiac team and was scheduled for a cardiac catheterization.

A cardiac catheterization was performed which did not show any narrowing or disease of vessels. His ejection fraction was 65% and the previous grafts and stent were patent. The cardiac team cleared the patient for discharge. The patient was scheduled for discharge, however he suddenly developed left-sided weakness and facial droop. NIH stroke scale was 9. The rapid response team was called and the patient underwent a CT scan with no contrast which showed no evidence of any acute or significant intracranial abnormality and no bleeding. Patient was transferred to the ICU and the neurology team was consulted. Neurology recommended the use of tPA as the patient was within the window period for use. While reviewing the guidelines and contraindications, it was discovered that the patient's aPTT was higher than the upper normal limit due to the recent use of unfractionated heparin for cardiac catheterization. At this time, the primary team made the decision to recheck the aPTT after one hour since the patient was still within the therapeutic window for tPA. The repeat aPTT was reported in the normal range, making the patient eligible for thrombolysis. The patient tolerated this intervention well and hemoglobin remained stable throughout hospital course. MRI was done 24 hours after the intervention showed evidence of acute ischemic changes involving the right anterior parietal lobe in the region of the right postcentral gyrus. On discharge, the patient's left-sided weakness and facial droop improved significantly. His NIH stroke scale was only 1 on discharge. The patient was discharged home in stable condition on aspirin and statin, as per neurology recommendations with close follow-up as an outpatient.

## 3. Discussion

According to the American Heart Association/American Stroke Association guidelines, the use of IV TPA is relatively contraindicated in patients who have received unfractionated heparin in the past 48 hours with an elevated activated partial thromboplastin time (aPTT). The apparent biological half-life of unfractionated heparin is approximately 60 to 90 minutes and is cleared via saturable dose dependent receptor triggered degradation and slower unsaturable renal based clearance mechanisms [4]. Higher doses including those above therapeutic levels carry a higher risk of adverse events including hemorrhagic complications given the increased reliance on renal clearance [4]. While the use of IV TPA in the setting of acute ischemic stroke within the community is often limited by the unknown onset of symptoms or presentation outside of the window period, stroke after cardiac catheterization provides a unique relatively controlled setting as most patients are hospitalized prior to intervention. A systematic multicentered review of 66 cases of acute ischemic strokes after cardiac catheterization by Khatri et al. (2008) showed 12 patients out of 66 with stroke were treated with revascularization approach and only 5 received IV thrombolysis alone. It demonstrated that patients who received thrombolysis in the setting of stroke after cardiac catheterization displayed a median improvement in NIHSS score from baseline to 24 hrs of 6 compared with 0 in the nonthrombolysis arm (*P* < 0.001). The median change from baseline to 7 days was 6.5 in those that received thrombolysis compared with 1.5 in those without intervention (*P* < 0.004) providing further evidence towards the benefit of early thrombolysis in this specific clinical setting [5].

In another retrospective study of 419 patients with stroke that underwent interventions, 14 of them were identified as having strokes during or immediately following cardiac catheterization. The study showed that 50% of the patients had favorable outcome [6].

While the risk of hemorrhagic complications including intracerebral hemorrhage (ICH) remains to be a potentially devastating complication following the use of thrombolysis, there were no reported cases of ICH within the thrombolysis arm in this systematic review by Khatri et al. [5]. While this review did incorporate a multicentered approach, it would be beneficial to incorporate a large sample size in future studies assessing the efficacy and safety of thrombolysis following SCC.

## 4. Conclusion

In summary, this case is of a patient who underwent cardiac catheterization and subsequently developed an ischemic stroke. The patient was successfully managed by use of tPA, however due to the recent use of unfractionated heparin closer monitoring of aPTT was required. There was a significant clinical improvement following the use of thrombolysis in our patient, an important aspect of the case was the repeat monitoring of the aPTT. Although our patient did not initially appear to be a candidate for thrombolysis, the short half-life of unfractionated heparin as discussed should be taken into account and this relative contraindication should be reassessed throughout the window period for thrombolysis.

Currently the use of thrombolysis in the setting of stroke after cardiac catheterization appears to be both case and institution based, and further studies are required to better understand the safety and efficacy of this treatment approach to allow for the creation of treatment protocols regarding this specific intervention.

## Abbreviations

aPTT: Activated partial thromboplastin time
CV ICU: Cardiovascular intensive care unit
IA: Intra-arterial
ICH: Intracranial hemorrhage
IV: Intravenous
NIHSS: National Institutes of Health Stroke Scale
PCI: Percutaneous coronary intervention
SCC: Stroke after cardiac catheterization
tPA: Tissue plasminogen activator.
